# Influence of Different Modalities of Grape Withering on Volatile Compounds of Young and Aged Corvina Wines

**DOI:** 10.3390/molecules25092141

**Published:** 2020-05-03

**Authors:** Davide Slaghenaufi, Anita Boscaini, Alessandro Prandi, Andrea Dal Cin, Vittorio Zandonà, Giovanni Luzzini, Maurizio Ugliano

**Affiliations:** 1 Department of Biotechnology, University of Verona, Villa Lebrecht, via della Pieve 70, 37029 San Pietro, Cariano, Italy; davide.slaghenaufi@univr.it (D.S.); aleprandi96@libero.it (A.P.); giovanni.luzzini@univr.it (G.L.); 2 Masi Agricola, Via Monteleone, 26, Sant’Ambrogio di Valpolicella, 37015 Verona VR, Italy; anita.boscaini@masi.it (A.B.); andrea.dalcin@masi.it (A.D.C.); vittorio.zandona@masi.it (V.Z.)

**Keywords:** post-harvest, withering, on-vine, fruttaio, wine aroma, Corvina

## Abstract

Withering is a practice traditionally used in various regions to produce sweet or dry wines. During withering there is an increase in sugar content but also a modification in volatile compound profiles. Controlling metabolic changes through the dehydration process to obtain wines with desired characteristics is therefore a challenging opportunity. The effects of two different withering technologies, post-harvest or on-vine with blocked sap vessel flow, on the volatile profile of young and aged Corvina red wines was investigated. The results showed that modulation of wine aroma due to the withering process is associated with fermentative metabolites, such as esters, higher alcohols, and acids, as well as grape-related compounds such as C6 alcohols, terpenes and norisoprenoids. Significant differences were also found by comparing the two withering techniques. Post-harvest in a traditional “fruttaio” warehouse wines showed higher content of ethyl acetate, ethyl butanoate, β-citronellol and 3-oxo-α-ionol, whereas post-harvest withering on-vine increased β-damascenone in wines. The type of withering technique has an influence on the evolution of some aroma compounds during the aging of wine, among them linalool, (*E*)-1-(2,3,6-trimethylphenyl)buta-1,3-diene (TPB), n-hexyl acetate, ethyl acetate, ethyl 3-methylbutanoate, 3-oxo-α-ionol and β-damascenone.

## 1. Introduction

Valpolicella is a wine producing region characterized by the traditional practice of post-harvest withering for the production of dry and sweet red wines, among which Amarone is the most famous [1]. Valpolicella is located in the north-east of Italy close to Verona city, with Corvina, Corvinone and Rondinella grapes being the traditional varieties employed for local wines [1]. At ripening, grapes are harvested and stored in a specific warehouse traditionally called a “fruttaio”, where they undergo slow dehydration [2]. The duration of withering varies depending on the wine type being produced, and it is generally monitored by assessing grape weight loss. In the case of Amarone or Recioto, withering generally lasts 2–3 months, with a weight loss of approximately 30% of the initial weight [3]. In the case of other wines such as Valpolicella Classico superiore as well as different IGT wines, a milder withering is usually carried out, lasting 4–8 weeks with weight loss of 10–15%.

Grape withering has a deep impact on the formation of the characteristic aroma of Amarone wine [4,5]. During the withering process, an increase in sugar content due to water loss is not the only transformation taking place. Phenolic and aromatic composition of grapes and wines is also affected [6,7,8,9,10,11,12], and skin wall composition as well as grape mechanical properties are also modified [13,14]. Interestingly, some of these processes are not due to dehydration but are the result of ongoing metabolic activities in the berry, resulting in peculiar gene expression patterns contributing to changes secondary metabolism [15,16,17].

Different grape withering techniques have been developed in the past, depending to local environment, grape variety and technical issues, entering in the local tradition and history as community heritage [18]. The different techniques can be divided into natural, forced and on-vine withering [18]. An example of a natural withering technique is the exposure of grapes to the sun, while in the forced method, grape dehydration is obtained using ventilated rooms like in the case of a modern fruttaio where withering conditions like temperature, humidity and air flow are controlled. On-vine withering can be obtained by practicing late harvest, cane cutting, or peduncle twist [18]. Though withering in Valpolicella is traditionally made in a fruttaio, there is an increasing interest to explore other postharvest methods to support traditional practices, in particular for mild withering processes requiring a short duration.

The aim of this research was to evaluate the effect of the two withering systems, in “fruttaio” and on-vine with peduncle twist, on the volatile profile of wines. The results were compared with those of wines obtained from not-withered grapes. Wines were also assessed after a period of aging, to evaluate the influence of the different withering practices on aging patterns of the resulting wines.

## 2. Results

### 2.1. Volatile Compounds in Young Wines

A total of 53 volatile compounds have been identified and quantified in wine samples (Table 1), including five alcohols, 3 C_6_ alcohols, 10 esters, three acids, 18 terpenes, seven norisoprenoids, seven benzenoids. The analysis of the variance (ANOVA) made between the three modalities, showed statistically significant (*p* < 0.05) differences for 35 compounds. Wines from withered grapes in fruttaio and on-vine were characterized by higher content in terpinen-4-ol, β-citronellol, 1,4-cineole, 3-oxo-α-ionol, vinylguaiacol, ethyl acetate, benzyl alcohol and ethyl vanillate. At the same time, samples fruttaio and on-vine compared to control showed lower amounts of 1-pentanol, 1-hexanol, *cis*-3-hexenol, *trans*-3-hexenol, isoamyl acetate, *n*-hexyl acetate, phenylethyl acetate ethyl 3-methylbutanoate, ethyl hexanoate, ethyl octanoate, hexanoic acid, octanoic acid, *cis*-linalool oxide and β-damascenone. Compared to each other, statistical differences of the two withering techniques were observed for 19 volatile compounds, among them 1-butanol, 1-hexanol, *trans*-3-hexenol, ethyl butanoate, β-citronellol, limonene, α-phellandrene, terpinolene, benzyl alcohol and methyl vanillate were found in higher concentration in fruttaio samples, while on-vine samples showed higher content of 2-butanol, ethyl acetate, octanoic acid, β-damascenone, TDN and 4-vinyl guaiacol.

Eighteen glycosidically bound compounds have been quantified (Table 2). The ANOVA showed significant differences for eight of these compounds. Compared to control, fruttaio samples and on-vine showed significantly lower concentrations in *cis*-3-hexenol, benzyl alcohol precursor and higher content of methyl vanillate and ethyl vanillate precursors. Comparing fruttaio and on-vine samples, statistically significant differences have been observed for six glycosidically bound compounds. Fruttaio samples were richer in 1-hexanol, *trans*-3-hexenol, geraniol and vanillin precursors, while on-vine samples showed higher concentration only for the phenylethyl alcohol glycosidically bound precursor. 

**Figure 1:**
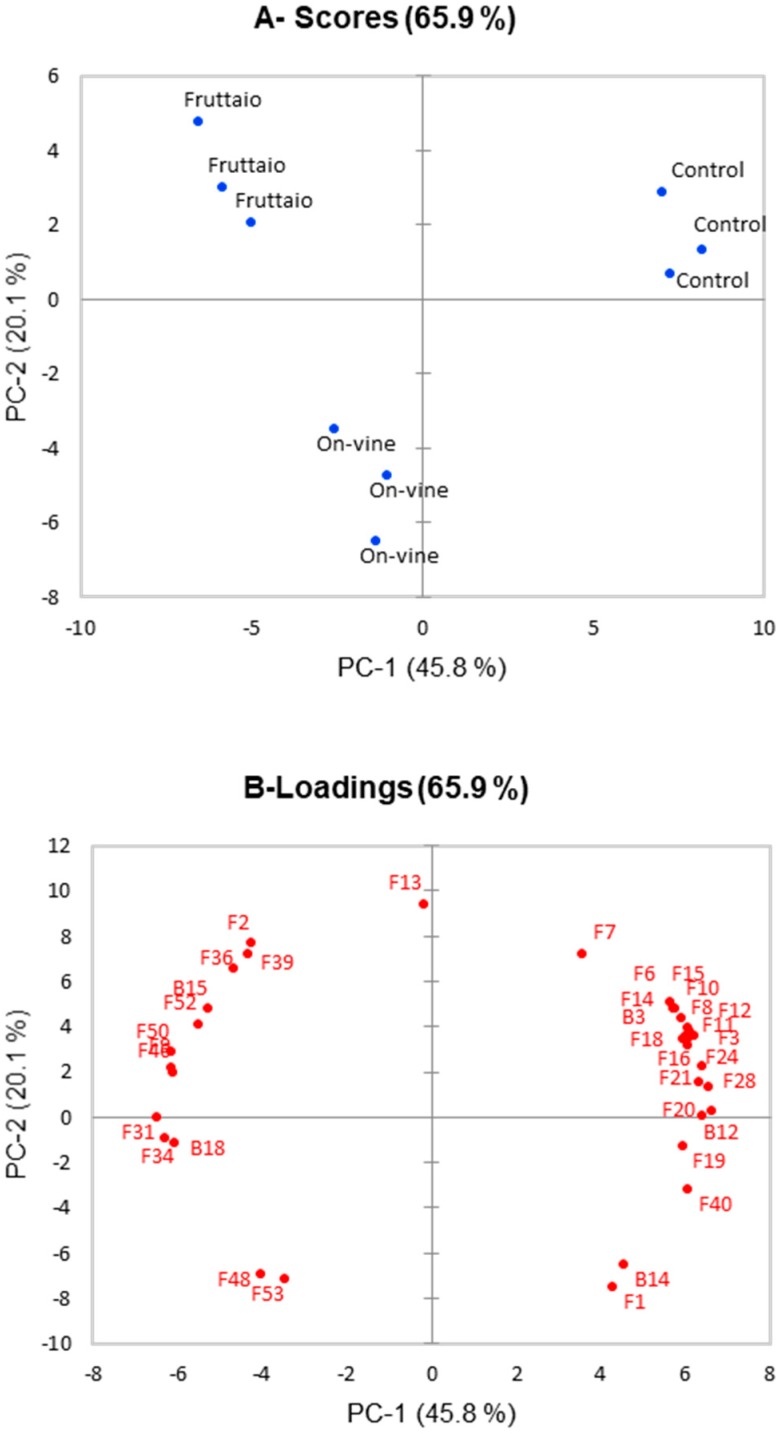
Principal component analysis showing aged wine samples scores (**A**) and loadings (**B**), only loadings with score value > 0.75 were shown. Loadings plot number correspond to: (F1) 2-Butanol, (F2) 1-Butanol, (F3) 1-Pentanol, (F6) 1-Hexanol, (F7) *trans*-3-Hexenol, (F8) *cis*-3-Hexenol, (F9) Ethyl acetate, (F10) Isoamyl acetate, (F11) n-Hexyl acetate, (F12) Phenylethyl acetate, (F13) Ethyl butanoate, (F14) Ethyl 3-methylbutanoate, (F15) Ethyl hexanoate, (F16) Ethyl octanoate, (F18) Ethyl lactate, (F19) 3-Methylbutanoic acid, (F20) Hexanoic acid, (F21) Octanoic acid, (F24) Linalool, (F28) α-Terpineol, (F31) β-Citronellol, (F34) 1,4-cineole, (F36) Limonene, (F39) Terpinolene, (F40) β-Damascenone, (F46) 3-oxo-α-Ionol, (F48) 4-Vinyl guaiacol, (F50) Benzyl alcohol, (F52) Methyl vanillate, (F53) Ethyl vanillate, (B3) bound cis-3-Hexenol, (B12) bound Benzyl alcohol, (B14) bound Phenylethyl alcohol, (B15) bound Vanillin, (B18) bound Ethyl vanillate.

Principal component analysis (PCA) showed that 65.9% of the total variance was explained by the first and second component (Figure 1). In fact, first principal component (PC-1) explained 45.8% of the total variance, while PC-2 explained 20.1%. Samples were separated into three clusters according to their withering technique. PC-1 mostly discriminated withered samples from not-withered. Control samples were characterized principally by esters, fatty acids, C_6_ compounds 1-hexanol, *trans*-3-hexenol, and *cis*-3-hexenol precursor. Instead withered compounds were characterized by terpenes like β-citronellol, linalool, terpinen-4-ol, 1,4-cineole; by the norisoprenoid 3-oxo-α-ionol; and by several benzenoids like phenylethyl alcohol, benzyl alcohol, methyl vanillate and its glycosidic precursor. PC-2 permitted to discriminate on-vine from fruttaio samples, the major drivers of this diversity were the ethyl butanoate, ethyl decanoate, ethyl vanillate, and the bound precursor of geraniol. 

### 2.2. Volatile Composition of Aged Wine

**Figure 2:**
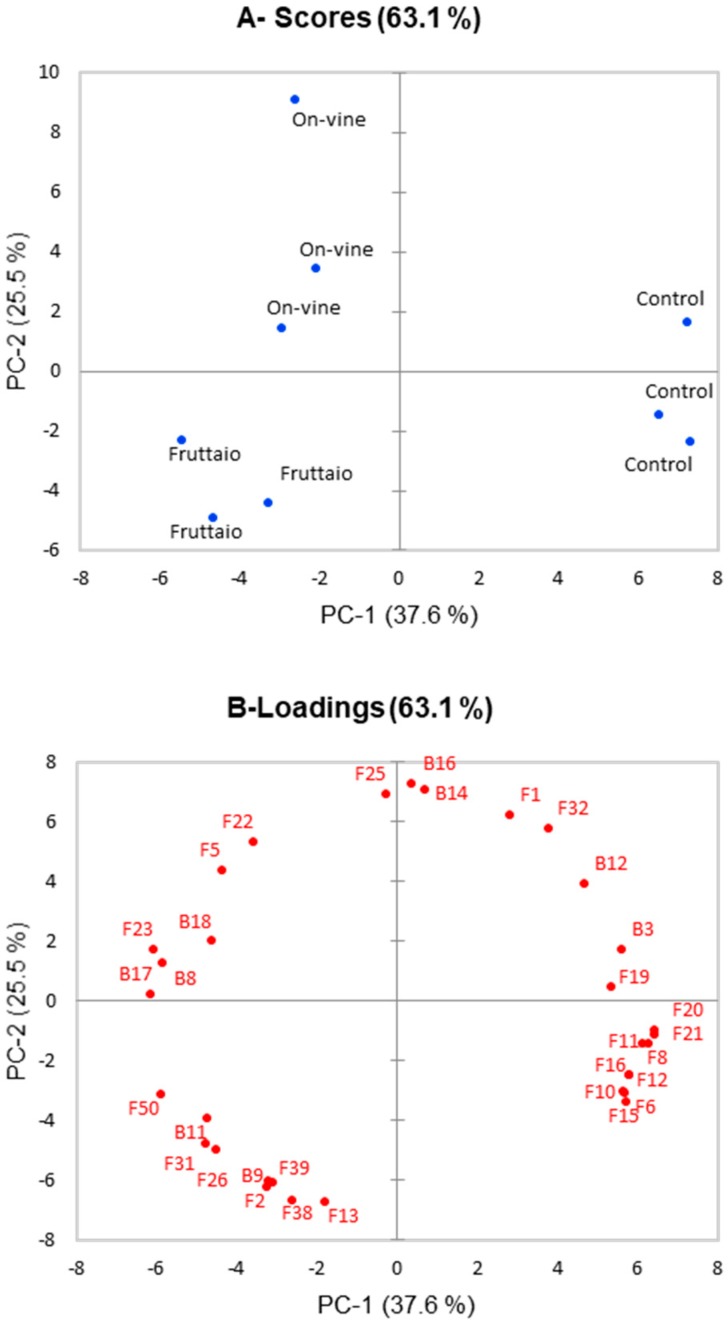
Principal component analysis showing aged wine samples scores (**A**) and loadings (**B**), only loadings with score value >0.75 were shown. Loadings plot number correspond to: (F1) 2-Butanol, (F2) 1-Butanol, (F5) Phenyl ethyl alcohol, (F6) 1-Hexanol, (F8) *cis*-3-Hexenol, (F10) Isoamyl acetate, (F11) n-Hexyl acetate, (F12) Phenylethyl acetate, (F13) Ethyl butanoate, (F15) Ethyl hexanoate, (F16) Ethyl octanoate, (F19) 3-Methylbutanoic acid, (F20) Hexanoic acid, (F21)Octanoic acid, (F22) *cis*-Linalool oxide, (F23) *trans*-Linalool oxide, (F25)Terpinen-1-ol, (F26) Terpinen-4-ol, (F31) β-Citronellol, (F32) p-Menthane-1,8-diol, (F38) p-Cymene, (F39) Terpinolene, (F50) Benzyl alcohol, (B3) bound *cis*-3-Hexenol, (B8) bound α-Terpineol, (B9) bound β-Citronellol, (B11) bound Geraniol, (B12) bound Benzyl alcohol, (B14) bound Phenylethyl alcohol, B16) bound 3-oxo-α-Ionol, (B17) bound Methyl vanillate.

After model aging, wines were different for 28 volatile compounds (Table 3) and seven bound compounds (*p* < 0.05) (Table 4). The fruttaio and the on-vine samples showed significant differences (*p* < 0.05) for 17 compounds, three of which were glycosidically bound precursors. The PCA analysis (Figure 2) after wine model aging showed a total variance of 63.1%. Three clusters were formed corresponding to the three conditions studied: control, fruttaio and on-vine. The compounds that most characterized these three groups were basically the same that were obtained in wine samples before model aging: the class of esters, acids and alcohols for control samples, terpenes and benzenoids for withered samples. 

## 3. Discussion

Post-harvest withering plays a central role in determining the compositional and sensory characteristics of Valpolicella red wines [1,3]. From a quantitative point of view, the main physiological change associated with this traditional practice is water loss, that is carried out up to an average of 30% weight loss depending on wine style. This has major implication for grape composition, most notably increased concentration of metabolites such as sugars, phenolics, and certain aroma compounds, directly influencing composition of the resulting wine. Additional important consequences of increased sugar levels are related to changes in yeast metabolism, which can further impact wine composition. However, it has been recently shown that post-harvest withering is not simply a dehydration process, with many complex metabolic transformations beyond simple concentration taking place inside the berry, inducing important modifications in the pool of grape secondary metabolites, including volatile compounds [17]. In consideration of this complex scenario, one of the purposes of the present study was to investigate how and to what extent withering of the grapes affects the volatile composition of the resulting wine. Second, and most important, this study had the objective to assess the potential of an alternative withering approach to modulate Corvina wine volatile composition. Although post-harvest withering is traditionally carried out in warehouses (locally called ”fruttaio”), there is an ongoing interest towards the exploration of alternative strategies that can be applied to obtain a suitable degree of over-ripening or withering, also with the aim of producing alternative wine types and styles [2]. Among these, cane-cut on-vine has been shown to positively influence wine aroma and phenolic composition [6,7,8,9,10,11,12]. In the present study, an alternative approach to on-vine withering, still based on blocking xylem flow but not involving cane cutting, was investigated in comparison with conventional fruttaio withering. As sugar levels at grape crush were similar for both withering modalities, any difference is expected to result from differences in grape composition in terms of secondary metabolites or interaction with yeast. 

### 3.1. Influence of Grape Withering on Volatile Composition of Corvina Wines 

**Figure 3:**
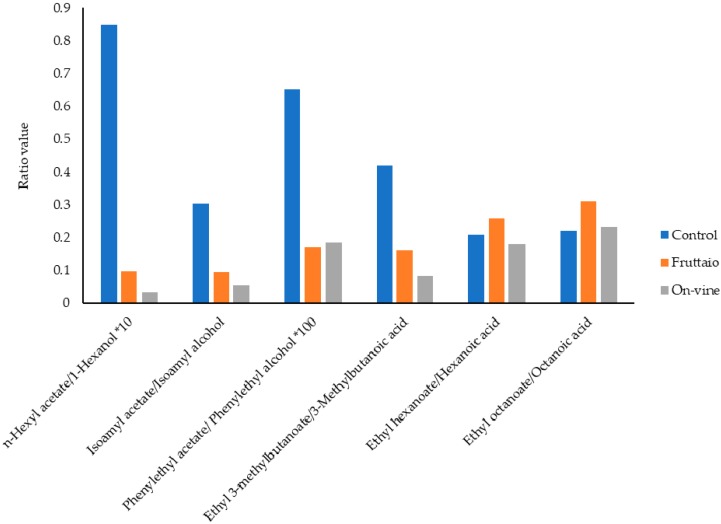
Ratio between esters and related precursors.

Analysis of free and glycosidically-bound volatile compounds of the wines at bottling showed that withering of the grapes significantly affected wine aroma compounds, influencing the concentrations of various classes of volatiles. Free compounds can have a direct influence on wine aroma while the bound compounds can act as an aroma reservoir that is released during aging. At a general level, fermentation-derived volatiles such as esters, higher alcohols, and acids, as well as grape-related compounds such as certain norisoprenoids, were mostly associated with non-withered grapes, whereas withering resulted in higher wine content in terpenes and benzenoids (Figure 1 and Table 1). Among compounds known to impact red wine aroma, acetate esters (i.e., isoamyl acetate) and ethyl fatty acid esters (i.e., ethyl hexanoate and octanoate) were strongly influenced by withering, which resulted in a significant decrease in the concentration of nearly all the analyzed esters. Esters are related to red wine’s fruity character [23] and are formed during alcoholic fermentation involving amino acid metabolism in the case of acetates, and fatty acid metabolism for ethyl esters [24]. The production of esters by yeast is influenced by several factors, and different studies have reported an influence of grape maturity [25] and levels of withering [4,9,26] on wine ester content, suggesting and influence of must sugar content on ester production. In agreement with these reports, wines from withered Corvina grapes, which at harvest displayed additional 2 Brix compared to control grapes, showed lower ester content. In particular, acetate esters were more impacted, in spite of the fact that higher alcohol content, a precursor of acetates, was not so different across treatments and in some cases was even greater in withered samples. Ester/alcohol ratios were calculated to establish esterification rates of the different esters, and in the case of acetates it appeared clear that acetylation was much higher in fermentation of non-withered grapes (Figure 3). Likewise, a higher acetylation rate was also observed for the control wine in the case of the ethyl ester of the branched chain fatty acid 3-methylbutanoic acid, also derived from amino acid metabolism. Conversely, although concertation of ethyl esters was higher in control samples, esterification of the corresponding fatty acid was similar in all treatments, so that it can be inferred that wine ester levels were determined by concentration of the corresponding fatty acid. It can be therefore assumed that, under our experimental conditions, withering impact on esters was due on one hand to reduced acetylation and on the other hand to reduced production of fatty acids, which would be in agreement with the observations of Saerens et al. (2008) [27]. In the case of ethyl fatty acid esters, the reduced availability of short chain fatty acid precursors could be due to the greater availability of unsaturated fatty acids in musts from withered grapes [28], which would result in reduced medium chain fatty acids biosynthesis [29]. An increase in available lipids can also reduce the expression of the *ATF1* gene and therefore lower acetyl transferase activity catalyzing the acetylation reaction [30]. Interestingly, ethyl acetate showed a completely different trend, its concentration increasing significantly in wines from withered grapes. Although acetyl transferase activities are expected to play a role in ethyl acetate formation by *S. cerevisiae*, recent observations indicated that acetyl transferases other than Atf1 and Atf2 contribute significantly to production of this ester [31], which could explain its different response to withering. 

C_6_ alcohols were also found to discriminate, with a high level of significance, control wines from the two withering modalities. C_6_ alcohols contribute to the “leafy” and “herbaceous” odors of wines [32]. In control samples *cis*-3-hexenol showed and odor active value (OAV, calculated as ration between concentration and odor threshold) higher than one therefore potentially contributed to wine aroma (OAV = 1.3). Instead, in withered samples, the C_6_ alcohols had OAV values lower than one. C_6_ alcohols are formed during berry crushing by enzymatic oxidation of grape unsaturated fatty acids, initiated by grape lipoxygenase enzymes [33]. Zenoni et al. (2016) [17] reported a decrease in the expression of lipoxygenase genes during withering of Corvina, which could explain the decrease in C_6_ alcohols observed here. However, other studies indicated an opposite trend [8,16], an increase in C_6_ aldehydes and alcohols during postharvest grape dehydration of Malvasia grape was reported [8], suggesting that more complex patterns could occur. 

Various terpenes were affected by withering, although trends varied depending on the specific molecule. The importance of monoterpene alcohols and cyclic terpenes to Corvina wines aroma was recently described, in particular for linalool [34,35]. In the present study, linalool was the main monoterpene alcohol detected and its concentration was significantly decreased by withering, in agreement with previous findings [17,36]. Considering that in control wines linalool had an OAV = 1.1, a possible contribution to wine aroma characteristics can be expected, whereas in wines from withered grapes this was not the case (OAV = 0.59 and 0.66 for fruttaio and on-vine respectively). Terpenes are produced in grapes through both the 1-deoxy-d-xylulose-5-phosphate/methylerythritol phosphate (DOXP/MEP) pathway and the mevalonic acid (MVA) pathway. In Corvina the influence of withering on these pathways is complex, with upper steps of the pathway being downregulated but late biosynthetic steps upregulated [16]. In addition to free forms of terpenes, grapes also contain non-volatile glycosylated forms of these compounds, which in Corvina can contribute significantly to terpenes level in finished through enzymatic and chemical hydrolysis during vinification [35,37]. Although in the present study differences in glycosidically-bound terpenes in the finished wines were relatively small, we observed generally higher concentrations of bound terpenes in withered wines (Table 2). Contrary to linalool, citronellol, the second most abundant monoterpene alcohols detected, increased with withering. In non-aromatic grapes such as Corvina, formation of citronellol is connected to the ability of yeast to reduce available geraniol including the portion derived from hydrolysis of geraniol glyosidic precursors [37,38]. Bound geraniol in finished wines increased in one of the withering modalities, supporting a possible contribution of bound geraniol to free citronellol levels. In wines from withered Pinot noir, Moreno et al. (2008) [9] also observed increased wine citronellol content. Among other terpenes, withering was consistently associated with increased contents of linalool oxides, limonene and 1,4-cineole. Small increases in the content of terpinen-4-ol in wine were also observed with withering, in agreement with the observations of Zenoni et al. (2016) [17]. The contribution of linalool oxides, limonene, 1,4-cineole and terpinen-4-ol to wine aroma seemed to be limited because their concentrations were found to be lower than the respective odor thresholds. 

Comparted to terpenes, norisoprenoids, were affected to a smaller extent by withering, with concentration of the potent odorant β-damascenone decreasing. The formation of this compound during winemaking is associated with multiple pathways involving acid- or yeast-mediated hydrolysis of different precursors [39,40]. The negative influence of withering on damascenone wine content could be due to complex factors and requires further investigation, also considering that other norisoprenoids such as 3-oxo-α-ionol had an opposite behavior and were found in higher concentrations in withered samples.

The benzenoids ethyl vanillate, and benzyl alcohol were found in higher concentrations in withered samples, unlike previously reports by Bellincontro et al. (2016) [4], albeit with more intense withering. 

Considering that wines made from withered grapes are generally destined to age, the volatile profile evolution of withered and non-withered samples was investigated by means of a model aging protocol [34]. Data showed that after model aging, the three sample modalities showed differences for a smaller number of compounds compared to young wines. Variations in compound concentrations are reported in Table 5. Esters remained one major factor discriminating wines from withered grapes after aging (Table 3 and Figure 2), with control wines exhibiting higher levels of ethyl fatty acids and acetate esters. Trends during aging were, however, different, with withered wines typically showing reduced losses of even increases in some cases compared to control. Esters can be formed or degraded according to wine pH and the ester/acid ratio. As a consequence, esters produced in a higher amount by yeast during fermentation, such as isoamyl acetate, tend to decrease during aging while branched-chain fatty acid esters increase [41,42,43]. Considering that the pH of the different samples was similar, we can conclude that by reducing esters formation during fermentation, withering resulted in reduced ester losses. One exception to this was observed for ethyl hexanoate, for which the rate of hydrolysis was similar in all treatments.

Aging patterns of certain terpenes also showed differences that could be associated with withering. For example, linalool in control wines during aging decreased from being sensorially active (OAV = 1.1) to an OAV < 1. Instead, in withered wines, linalool concentration tended to increase with aging. Particularly in the Fruttaio samples, after aging linalool had an increase of 8.3 µg/L, 1.5 times higher than young Fruttaio wines. This increase could be due to a higher content of glycosylated precursors in withered samples. However, the analysis of bound compounds in young wines did not show significant differences between samples that could explain the observed differences. It may be that different precursors forms of linalool exist in our samples that were not quantified with the employed method. *cis*-Linalool oxide increased more markedly in wines from withered grapes, and this could be attributed to acid hydrolysis of glycosidic precursors (diendiol) [44], and 3,7-dimethyloct-1-ene-3,6,7-triol (triol) [45]. This last pathway seemed more consistent in this sample set, as both *cis-* and *trans*-linalool oxide bound precursors did not decrease with aging. 1,8-Cineole also displayed substantially different behaviors during aging between wines from withered and non-withered grapes, with concentration increasing during aging only in withered wines. This could be due to the fact that young wines from withered grapes exhibited significantly higher content of tepinen-4-ol, which we have recently shown to be a precursor to 1,8-cineole in Corvina wines [34]. 

**Figure 4:**
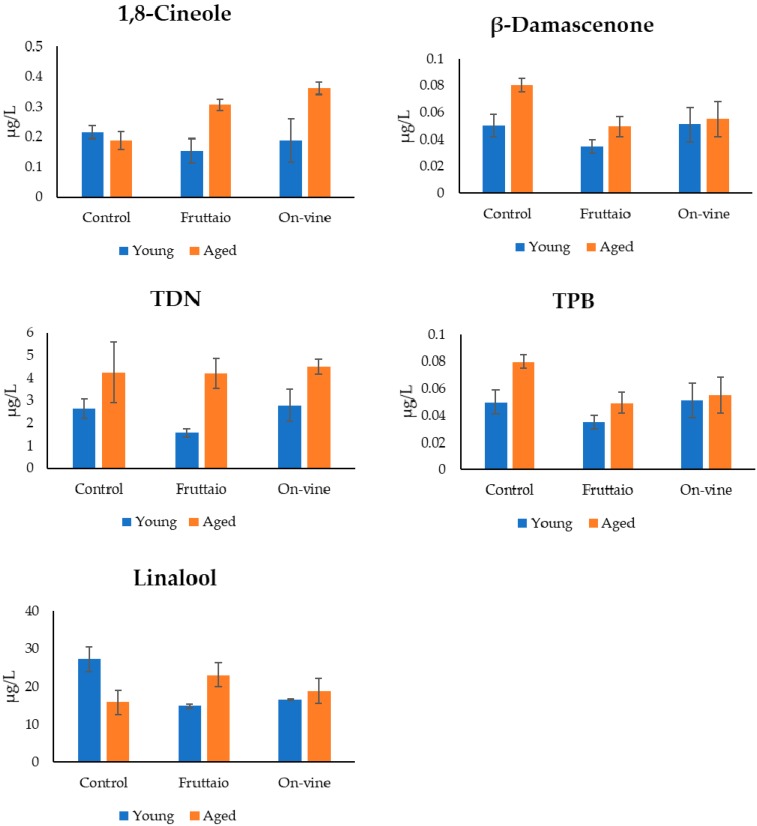
1,8-Cineole, β-damascenone, (*E*)-1-(2,3,6-trimethylphenyl)buta-1,3-diene (TPB),1,1,6-Trimethyl-1,2-dihydronapthalene (TDN) and linalool content in young and model aged wines.

β-Damascenone evolution with aging also highlighted a major difference associated with withering. Evolution of β-damascenone in Corvina wines during aging is characterized by a complex trend with an initial increase followed by a decline [34], reflecting simultaneous release from precursors (until available) followed by degradation through various reactions [46,47]. In the present study, the concentration of β-damascenone after model aging remained stable in control wines, and increased in withered samples reaching, in the case of on-vine samples, the level of control (Figure 4). Samples withered in fruttaio showed the most important increase of β-damascenone.

The (*E*)-1-(2,3,6-trimethylphenyl)buta-1,3-diene (TPB) also showed interesting differences in aged wines. A significant important increase has been observed after aging only in control samples. A slight increase occurred in samples withered in fruttaio while on-vine samples did not show any changes with aging. TPB has a tobacco aroma at low concentrations and geranium like odor at higher concentrations [48]. Its concentration in Corvina wine has been correlated with wine ageing [34]. It has been suggested that in red wine rich in tannins, TPB could react with polyphenols, resulting in a lower concentration like in Shiraz and Cabernet Sauvignon (Janusz 2003). It is reported that withered wines have a higher polyphenol content [10,12] while Corvina is known to be poor in polyphenols, so a TPB–tannin reaction may explain the lower content found in aged withered samples. 

TDN variation was higher in fruttaio samples; however, it should be noticed that at the end of model aging samples of all the three modalities, it reached the same concentration level. TDN has a kerosene-like aroma, the concentration in wine was reported to be influenced by grape sun exposure, wine age, pH, and storage temperature [49,50]. Our data suggested that TDN concentration in wine was not affected by grape withering, the level of TDN formed in withered wine could depend on the TDN precursors accumulated just before the start of the withering process, harvest or peduncle twist. 

The occurrence of aroma notes related to TDN is often associated with aged wines; however, in the control and on-vine samples, an OAV of 1.32 and 1.39, respectively, was observed already in the young wine, indicating a possible sensory contribution. 

### 3.2. Influence of Withering Modality on Volatile Composition of Corvina Wine

In comparison with the differences due to withering, those associated with the withering modality were quantitatively less important and restricted to a small number of volatiles. In young wines, small but statistically significant increases in the concentration of different wine esters and citronellol were observed in fruttaio withering compared to on-vine withering, whereas linalool, β-damascenone, and α-ionol were mostly associated with on-vine withering. The trends observed for glycosylated volatiles were different, as in this case fruttaio wines exhibited a higher content of bound terpenes such as geraniol and nerol, whereas benzenoids and certain norisoprenoids were more abundant in on-vine withering. Several studies have investigated the influence of on-vine over-ripening or even on-vine withering on grape composition, but only in a few cases these were compared with other methods of withering. Zamboni et al. (2008) [16] provided interesting insights in differences existing at a transcriptomic level between on-vine and off-vine (fruttaio) withering of Corvina, indicating that differences in transcripts associated with secondary metabolites were minor [16]. However, on-vine withering did not involve any blockage of vascular tissues, so results are hard to compare with the present study. 

Interestingly, some differences between the two withering modalities could be observed after aging. For example, the above-mentioned trend of reduced ester loss was lower in the case of on-vine withering, to the point that some esters actually increased during aging of wines from on-vine withering. Increases in certain grape-derived compounds were also dependent on withering, as in the case of 3-oxo-α-ionol, p-menthane-1,8-diol. Overall, it appeared that the two different withering conditions induced similar types of changes, mostly modulating the extent of such changes. 

## 4. Materials and Methods 

### 4.1. Chemicals

Octan-2-ol (97%), 1-hexanol (99%), *cis*-3-hexenol (98%), *trans*-3-hexenol (97%), vanillin (99%), 2,6-dimethoxyphenol (99%), linalool (97%), terpinen-4-ol (≥95%), α-terpineol (90%), nerol (≥97%), geraniol (98%), linalool oxide (≥97%), β-citronellol (95%), p-cymene (99%), terpinolene (≥85%), γ-terpinene (≥97%), limonene (97%), 1,8-cineole (99%), 1,4-cineole(≥98.5%), β-damascenone (≥98%), isoamyl alcohol (98%), benzyl alcohol (≥99%), 2-phenylethanol (≥99%), ethyl acetate (99%), ethyl butanoate (99%), ethyl 3-methyl butanoate ((≥98%), isoamyl acetate (≥95%), ethyl hexanoate (≥95%), phenylethyl acetate (99%), n-hexyl acetate (≥98%), ethyl lactate (≥98%), ethyl octanoate (≥98%), ethyl decanoate (≥98%), hexanoic acid (≥99%), octanoic acid (≥98%), α-phellandrene (95%), p-menthane-1,8-diol (97%), 3-methylbutanoic acid (99%), α-ionone (90%), 1-pentanol (99%), 1-butanol (≥99%), 2-butanol (≥99%), ethyl guaiacol (≥99%), vinyl guaiacol (≥98%), methyl-vanillate (99%) and ethyl vanillate (99%), were supplied by Sigma Aldrich (Milan, Italy). Dichloromethane (≥99.8%) and methanol (≥99.8%), were provided by Honeywell (Seelze, Germany). Sodium chloride (≥99.5%) was supplied by Sigma Aldrich (Milan, Italy).

### 4.2. Wine Samples

**Figure 5:**
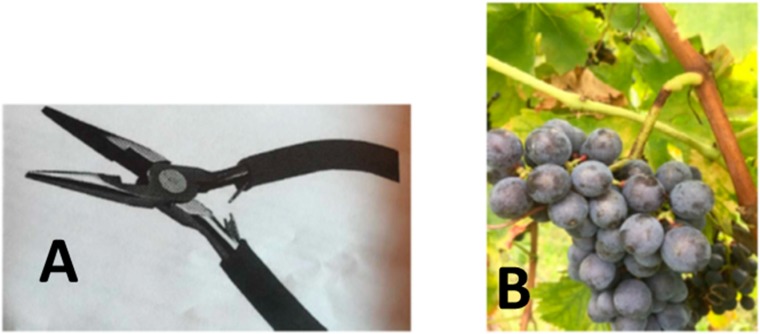
Plier for the peduncle twist (**A**), and grape after peduncle twist (**B**). Photo courtesy of Masi.

Wine samples were produced in the experimental facility of Masi Agricola. Corvina grapes from the 2017 vintage were obtained from a single 3 ha vineyard (45°29′22.9″ N 10°46′20.5″ E) located in the town of Lazise, 25 km west of Verona. The vineyard site was flat, with an altitude of 70 m asl. Vines had 12 years of age and were trained with a double arch cane system, with an average of 60,000 gems/ha and a yearly production of 11–12 tons/ha. Upon achievement of a sugar level of 21 Brix (27 of September), three experimental modalities were applied. Control grapes were hand harvested, placed in 7 kg harvest bins and transferred to the experimental winery where they were directly vinified as described later. A second batch, labelled “fruttaio”, was harvested on the same day and the harvest bins were placed in a non-conditioned withering warehouse until November 4, when the berries had achieved a sugar content of 23.2 °Brix. Average conditions in the warehouse over the same period for the previous 10 years indicated a gradual temperature decrease (from 16 °C to 7 °C) and a progressive increase in relative humidity (from 55% to 80%). A third modality, labelled “on-vine”, was obtained by applying a peduncle twist in order to block vascular tissue and induce grape dehydration (Figure 5). Weather conditions in the vineyard area during the on-vine withering period were obtained from the Arpa Veneto meteorological database (http://www.arpa.veneto.it/). These conditions were in line with the typical conditions of the area, and were as follows: average daily minimum temperature of 7.5 °C), average daily maximum temperature of 20.6 °C, average daily mean temperature of 13.1 °C, average daily minimum relative humidity 44%, average daily maximum relative humidity of 97%, total precipitations 19 mm (1 rainy day). Upon achievement of a sugar content of 23.2 Brix (25 October), grapes were hand harvested as for the other modalities and were vinified. All vinifications were carried out in triplicate. For each vinification, 100 kg of grapes were destemmed and crushed, and the obtained musts were added with 100 mg/L of potassium metabilsulfite. Fermentations were conducted 75 L steel tanks by inoculation with the the proprietary *S. cerevisiae* yeast MASY03 (Microbion, Castel d’Azzano, Italy). At the end of fermentation, potassium metabisulphite was added in order to reach 30 mg/L of free SO_2_, wines were then filtrated at 1 micron and bottled. Sample bottles were stored at 16 °C until analysis. Data concerning grapes at harvest and wine at bottling are summarized in Table 5.

### 4.3. Wines Model Aging

Model aging was carried out as described by Slaghenaufi et al. (2019) [35], by placing 115 mL of wine in glass vial and crimped leaving 0.8 mL of headspace corresponding to 2 mg/L of oxygen. Vials were then crimped and sealed with Araldite glue and stored at 40 °C for 12 weeks.

### 4.4. Volatile and Glycosidically-Bound Compound Analysis

Volatile and glycosidically-bound compounds have been analyzed as described by Slaghenaufi et al. (2019) [35] with minor modification. In total, 50 mL of sample was added with 20 μL of internal standard solution (2-octanol at 42 mg/L in ethanol) and diluted with 50 mL of distilled water. The solution was then loaded on a BOND ELUT-ENV, SPE cartridge, containing 1 g of sorbent (Agilent Technologies, Santa Clara, CA, USA), previously activated with 20 mL of methanol and equilibrated with 20 mL of water. The cartridge was then washed with 15 mL of water. Free volatile compounds were eluted with 10 mL of dichloromethane, and then concentrated under gentle nitrogen stream to 200 μL prior to GC injection. Bound compounds were recovered with 20 mL of methanol. Methanol was then evaporated under vacuum. Bound compounds were then dissolved in 5 mL of citrate buffer (pH 5). were added to dissolve bound compounds to that 200 μL of an enzyme preparation AR2000 (DSM, Brussels, Belgium, prepared at 70 mg/mL in citrate buffer) were added and incubated at 37 °C for 24 h under shaking (150 rpm). 

A calibration curve was prepared for each analyte using seven concentration points and three replicate solutions per point in model wine (12% *v*/*v* ethanol, 3.5 gr/L tartaric acid, pH 3.5) [51]. A total of 20 μL of internal standards 2-octanol (42 mg/L in ethanol), was added to the solution. SPE extraction and GC-MS analysis were performed as described above for the samples. Calibration curves were obtained using Chemstation software (Agilent Technologies) by linear regression, plotting the response ratio (analyte peak area/internal standard peak area) against concentration ratio (analyte added concentration/internal standard concentration). Method characteristics are reported in Table 6. The 3-oxo-α-ionol analysis was semi-quantitative and they were expressed as µg/L of 2-octanol equivalent (internal standard) as for this compound no commercial standard was available. 

Terpenoids have been analyzed by SPME-GC-MS as described by Slaghenaufi and Ugliano (2018) [34]. In total, 5 mL of wine added with 5 µL of internal standard solution (octen-2-ol at 420 mg/L in ethanol) was placed into a 20 mL vial, together with 5 mL of mQ water (18.2 MΩ-cm) and 3 g of NaCl. The sample was equilibrated for 1 min at 40 °C. Subsequently SPME extraction was performed using a 50/30 µm divinylbenzene–carboxen–polydimethylsiloxane (DVB/CAR/PDMS) fiber (Supelco, Bellafonte, PA, USA) exposed to sample headspace for 60 min at 40 °C. The fiber was then desorbed into the injector port of a HP 7890A (Agilent Technologies) gas chromatographer coupled to a 5977B mass spectrometer. Injection was performed at 250 °C for 5 min in splitless mode. Chromatographic separation was done using a DB-WAX capillary column (30 m × 0.25, 0.25 µm film thickness, Agilent Technologies). Helium was used as carrier gas at 1.2 mL/min of constant flow rate. The temperature of the GC oven was initially kept at 40 °C for 3 min, and then programmed to raise at 230 °C at 4 °C/min, maintained for 20 min. Mass spectrometer operated in electron ionization (EI) at 70 eV with ion source temperature at 250 °C and quadrupole temperature at 150 °C. Acquisition was done in Selected Ion Monitoring (SIM). Quantification was performed using calibration curve obtained by standards addition at 7 different concentration levels in Corvina wine. A total of 5 μL of internal standards 2-octanol (420 mg/L in Ethanol), 5 mL of water and 3 g of NaCl were added to 5 mL of standard solutions. GC-MS analysis was performed as described above for the samples. Linear term for calibration curves were obtained using Chemstation software (Agilent Technologies) by linear regression, plotting the response ratio (analyte peak area/internal standard peak area) against concentration ratio (analyte added concentration/internal standard concentration). The analysis of vitispirane, terpinen-1-ol, TPB, TDN, and ho-trienol was semiquantitative as no standards was available. Results for these molecules were expressed as µg/L of 2-octanol equivalent (internal standard) (Table 6).

### 4.5. Statistic 

Data treatment, ANOVA, Tukey post-hoc test and PCA were performed using XLSTAT 2017 (Addinsoft SARL, Paris, France).

## 5. Conclusions

The present study allowed to characterize the influence of post-harvest withering of Corvina grapes on the aroma profile of wines. The aromatic contribution given by the withering on-vine or in a traditional withering warehouse (fruttaio) was also been evaluated.

Withering resulted in a lower content in fermentation-derived volatiles such as esters, higher alcohols, and acids, as well as grape-related compounds such as C_6_ alcohols, and certain norisoprenoids like β-damascenone. Terpenes showed different behaviors according to the compound. Linalool, the major terpene found in the sample wines analyzed, and *cis*-linalool oxide were negatively influenced by the withering process while β-citronellol and 1,4-cineole showed a different trend and it was found in higher concentrations in withered samples. The same trend was observed for ethyl acetate, ethyl vanillate, benzyl alcohol and vanillin 

The aroma profile of wines obtained by whitering in fruttaio was characterized by higher concentrations of esters such as ethyl acetate compared to on-vine withering. Wines withered in fruttaio were also distinguished by higher concentrations of β-citronellol and 3-oxo-α-ionol, while on-vine withering showed higher content of β-damascenone. The withering process as well as the technique employed also influenced the behavior of compounds during aging, showing different variation. 

Overall, the results of the present study indicate that on-vine withering with blocked xylem is an interesting alternative to conventional fruttaio withering for the production of wines where a mild withering is requested. Although on-vine withering can only be carried out in years where climatic conditions are suitable, the possibility to explore this kind of withering technique is of interest to reduce the workload of fruttaio facilities and the energy cost associated with their functioning, reducing the environmental impact of the winemaking process.

## Figures and Tables

**Table 1:** Concentration (µg/L) of free compounds in young wine samples. Mean, standard deviation (SD) and ANOVA.

		Control		Fruttaio		On-Vine		
Compounds	Odor Threshold ^1^	Mean	SD	Mean	SD	Mean	SD	*p*-Value
**Alcohols**								
2-Butanol		3283.8 ^b^	±207.9	2203.1 ^a^	±80.3	3467.7 ^b^	±93.1	<0.0001
1-Butanol		269.7 ^a^	±6.7	506.3 ^b^	±48.8	228.0 ^a^	±48.3	0.000
1-Pentanol		58.3 ^b^	±5.3	43.0 ^a^	±1.5	42.8^a^	±1.4	0.002
Isoamyl alcool	30,000	35,372.3 ^a^	±1853.6	33,820.8 ^a^	±922.9	34,951.5 ^a^	±2051.8	0.541
Phenylethyl Alcohol	14,000	16,240.6 ^a^	±721.1	20,779.8 ^a^	±466.8	16,568.9 ^a^	±6073.6	0.290
**C6 Alcohols**								
1-Hexanol	8000	3292.5 ^c^	±171.9	2292.4 ^b^	±84.2	1951.1 ^a^	±70.7	<0.0001
*trans*-3-Hexen-1-ol		39.4 ^b^	±2.4	32.0 ^b^	±6.59	21.4 ^a^	±2.0	0.006
*cis*-3-Hexen-1-ol	400	520.3 ^b^	±26.9	92.7 ^a^	±13.2	65.5 ^a^	±7.3	<0.0001
**Acetate Esters**								
Ethyl acetate	12,000	19,537.4 ^a^	±1896.3	49,654.9 ^c^	±4413.3	33,649.2 ^b^	±7566.5	0.001
Isoamyl acetate	30	10,732.9 ^b^	±1503.1	3216.1 ^a^	±631.4	1877.3 ^a^	±293.0	<0.0001
n-Hexyl acetate	1800	279.3 ^b^	±41.8	22.3 ^a^	±1.5	6.47 ^a^	±1.09	<0.0001
Phenylethyl acetate	2400	106.2 ^b^	±11.0	35.3 ^a^	±4.5	30.7 ^a^	±1.9	<0.0001
**Ethyl Esters**								
Ethyl butanoate	20	254.7 ^b^	±14.7	265.6 ^b^	±28.1	210.1 ^a^	±16.4	0.036
Ethyl 3-methylbutanoate	3	215.4 ^b^	±32.5	64.6 ^a^	±14.1	36.7 ^a^	±6.3	<0.0001
Ethyl hexanoate	14	548.5 ^b^	±33.4	331.0 ^a^	±9.5	302.1 ^a^	±24.9	<0.0001
Ethyl octanoate	5	452.1 ^b^	±50.2	254.3 ^a^	±10.7	249.6 ^a^	±27.9	0.000
Ethyl decanoate	200	45.6 ^a^	±6.2	50.6 ^a^	±5.5	38.6 ^a^	±6.5	0.132
Ethyl lactate	100000	1087.9 ^b^	±49.6	690.6 ^a^	±34.7	647.8 ^a^	±64.2	<0.0001
**Fatty Acids**								
3-Methylbutanoic acid	250	511.9 ^b^	±38.5	402.3 ^a^	±6.6	449.1 ^a^	±19.0	0.005
Hexanoic acid	2080	2623.6 ^c^	±130.17	1286.5 ^a^	±103.3	1683.5 ^b^	±36.2	<0.0001
Octanoic acid	2560	2044.8 ^c^	±184.0	820.0 ^a^	±36.8	1066.7 ^b^	±39.8	<0.0001
**Terpenes**								
*cis*-Linalool oxide	3000	6.57 ^b^	±3.12	1.29 ^a^	±0.78	1.33 ^a^	±0.19	0.038
*trans*-Linalool oxide	6000	0.415 ^a^	±0.211	0.330 ^a^	±0.130	0.50 ^a^	±0.282	0.797
Linalool	25	27.3 ^b^	±3.3	14.8 ^a^	±0.5	16.5 ^a^	±0.2	0.000
Terpinen-1-ol		0.351 ^a^	±0.065	0.429 ^a^	±0.162	0.519 ^a^	±0.066	0.242
Terpinen-4-ol		0.092 ^a^	±0.021	1.163 ^b^	±0.200	0.795 ^b^	±0.173	0.026
Ho-trienol	110	0.060 ^a^	±0.010	0.047 ^a^	±0.010	0.075 ^a^	±0.043	0.478
α-Terpineol	250	13.9 ^b^	±1.3	6.54 ^a^	±0.09	7.09 ^a^	±0.30	<0.0001
Nerol	400	3.95 ^a^	±0.53	4.89 ^a^	±0.43	3.36 ^a^	±0.96	0.828
Geraniol	30	6.69 ^a^	±0.69	6.50 ^a^	±0.79	6.61 ^a^	±0.20	0.931
β-Citronellol	100	4.14 ^a^	±0.18	12.58 ^c^	±0.44	10.18 ^b^	±1.87	0.000
p-Menthane-1,8-diol		<LOQ		<LOQ		<LOQ		
α-Phellandrene		0.035 ^b^	±0.013	0.040 ^b^	±0.006	0.016 ^a^	±0.002	0.029
1,4-Cineole	0.54	0.110 ^a^	±0.013	0.205 ^b^	±0.023	0.182 ^b^	±0.024	0.003
1,8-Cineole	1.1	0.215 ^a^	±0.022	0.153 ^a^	±0.040	0.176 ^a^	±0.068	0.336
Limonene		0.228 ^a^	±0.028	0.388 ^b^	±0.060	0.225 ^a^	±0.040	0.006
γ-Terpinen		1.10 ^a^	±0.51	1.15 ^a^	±0.09	1.03 ^a^	±0.19	0.904
p-Cymene		0.083 ^a^	±0.008	0.145 ^a^	±0.077	0.093 ^a^	±0.021	0.289
Terpinolene		0.137 ^a^	±0.024	0.257 ^b^	±0.045	0.125 ^a^	±0.041	0.010
**Norisoprenoids**								
β-Damascenone	0.05	3.47 ^c^	±0.27	1.69 ^a^	±0.13	2.80 ^b^	±0.10	<0.0001
α-Ionone		2.27 ^b^	±0.70	0.85 ^a^	±0.34	1.03 ^a^	±0.67	0.050
α-Ionol		0.233 ^a^	±0.019	0.283 ^a^	±0.026	0.25 ^a^	±0.010	0.955
Vitispirane		<LOQ		<LOQ		<LOQ		
TPB		0.050 ^a^	±0.009	0.035 ^a^	±0.006	0.051 ^a^	±0.013	0.140
TDN	2	2.64 ^b^	±0.42	1.57 ^a^	±0.18	2.79 ^b^	±0.69	0.040
3-oxo-α-Ionol		1.58 ^a^	±0.24	2.65 ^c^	±0.20	2.10 ^b^	±0.23	0.003
**Benzenoids**								
4-Ethyl guaiacol	33	<LOQ		<LOQ		<LOQ		
4-Vinyl guaiacol	1100	7.36 ^a^	±0.44	9.40 ^b^	±0.53	11.63 ^c^	±1.63	0.006
2,6-Dimethoxyphenol		0.108 ^a^	±0.018	0.015 ^a^	±0.007	0.022 ^a^	±0.001	0.428
Benzyl Alcohol		128.3 ^a^	±4.9	229.1 ^c^	±15.1	173.1 ^b^	±24.7	0.001
Vanillin	200	2.13 ^a^	±0.84	2.04 ^a^	±0.95	0.888 ^a^	±0.672	0.204
Methyl vanillate		4.37 ^a^	±0.09	4.86 ^b^	±0.11	4.49 ^a^	±0.05	0.001
Ethyl vanillate		16.4 ^a^	±1.1	21.6 ^b^	±2.0	31.4 ^c^	±5.2	0.004

Values in the same row with different letters indicate statistically significant differences, *p* < 0.05; ^1^ Data from: Ferreira et al. (2000) [19], Francis et al. (2005) [20], Sacks et al. (2012) [21] and Antalick et al. (2015) [22]. <LOQ: Values below the limit of quantification.

**Table 2:** Concentration (µg/L) of glycosidically-bound compounds in young wine samples. Mean, standard deviation (SD) and ANOVA.

	Control		Fruttaio		On-Vine		
Compounds	Mean	SD	Mean	SD	Mean	SD	*p*-Value
**Alcohols**							
Phenylethyl alcohol	284.9 ^b^	±22.5	191.7 ^a^	±11.0	282.0 ^b^	±47.8	0.017
**C6 alcohols**							
1-Hexanol	203.8 ^b^	±15.8	216.5 ^b^	±29.8	159.6 ^a^	±17.5	0.043
*trans*-3-Hexen-1-ol	1.80 ^a^	±0.28	3.27 ^b^	±0.41	2.03 ^a^	±0.29	0.003
*cis*-3-Hexen-1-ol	25.4 ^b^	±2.4	12.2 ^a^	±1.6	11.0 ^a^	±1.6	0.000
**Terpenes**							
*cis*-Linalooloxide	1.93 ^a^	±0.71	2.15 ^a^	±0.53	2.06 ^a^	±0.64	0.909
*trans*-Linalooloxide	2.17 ^a^	±0.72	2.74 ^a^	±0.88	2.49 ^a^	±0.61	0.666
Linalool	5.71 ^a^	±1.40	6.82 ^a^	±0.82	6.91 ^a^	±0.94	0.378
Terpinen-4-ol	0.068 ^a^	±0.020	0.070 ^a^	±0.024	0.069 ^a^	±0.032	0.999
α-Terpineol	2.00 ^a^	±0.52	2.19 ^a^	±0.02	2.42 ^a^	±0.20	0.343
β-Citronellol	0.163 ^a^	±0.048	0.747 ^b^	±0.051	0.130 ^a^	±0.030	0.051
Nerol	15.1 ^a^	±0.6	16.4 ^a^	±1.8	11.3 ^a^	±4.0	0.120
Geraniol	20.7 ^a^	±1.7	24.1 ^b^	±1.4	19.0 ^a^	±1.1	0.013
**Norisoprenoids**							
3-oxo-α-Ionol	5.44 ^b^	±0.43	2.80 ^a^	±1.41	4.40 ^ab^	±1.56	0.102
α-Ionol	0.035 ^a^	±0.017	0.015 ^a^	±0.010	0.030 ^a^	±0.018	0.329
**Benzenoids**							
Vanillin	0.175 ^a^	±0.095	9.41 ^c^	±1.36	1.60 ^b^	±0.22	0.001
Methyl vanillate	4.01 ^a^	±0.21	5.17 ^b^	±0.29	4.92 ^b^	±0.02	0.001
Ethyl vanillate	0.365 ^a^	±0.129	0.737 ^b^	±0.209	0.747 ^b^	±0.188	0.065
Benzyl Alcohol	303.7 ^b^	±58.2	112.4 ^a^	±10.7	163.9 ^a^	±21.5	0.002

Values in the same row with different letters indicate statistically significant differences (*p* < 0.05). <LOQ: Value below the limit of quantification.

**Table 3:** Concentration (µg/L) of free compounds in aged wine samples. Mean, standard deviation (SD) and ANOVA.

	Control		Fruttaio		On-Vine		
Compounds	Mean	SD	Mean	SD	Mean	SD	*p*-Value
**Alcohols**							
2-Butanol	3019.8 ^b^	±237.4	1980.9 ^a^	±291.9	3276.2 ^b^	±112.6	0.001
1-Butanol	247.4 ^a^	±37.2	452.1 ^b^	±16.7	206.9 ^a^	±50.3	0.000
1-Pentanol	17.9 ^a^	±0.6	15.0 ^a^	±5.2	14.2 ^a^	±1.9	0.398
Isoamyl alcool	60,167.0 ^a,b^	±1467.0	55,052.5 ^a^	±5134.7	63,751.1 ^b^	±2291.3	0.051
Phenylethyl Alcohol	20,612.4 ^a^	±2721.8	25,084.0 ^ab^	±2609.2	28,513.4 ^b^	±4720.3	0.083
**C6 Alcohols**							
1-Hexanol	3199.2 ^c^	±22.3	2231.4 ^b^	±208.7	1787.8 ^a^	±102.2	<0.0001
*trans*-3-Hexen-1-ol	51.9 ^c^	±2.6	40.4 ^b^	±1.3	26.2 ^a^	±7.4	0.001
*cis*-3-Hexen-1-ol	609.6 ^b^	±7.7	110.0 ^a^	±39.5	74.0 ^a^	±11.1	<0.0001
**Acetate Esters**							
Ethyl acetate	19,178.22 ^a^	±7696.5	32,697.7 ^b^	±2026.1	38,187.8 ^b^	±5575.2	0.015
Isoamyl acetate	3732.0 ^b^	±188.2	1435.2 ^a^	±852.1	760.8 ^a^	±300.4	0.001
n-Hexyl acetate	82.8 ^b^	±3.1	8.9 ^a^	±2.1	8.0 ^a^	±2.8	0.000
Phenylethyl acetate	55.2 ^b^	±1.9	29.0 ^a^	±10.2	23.8 ^a^	±2.2	0.002
**Ethyl Esters**							
Ethyl butanoate	255.2 ^a^	±12.2	296.2 ^b^	±20.1	231.7 ^a^	±24.2	0.018
Ethyl 3-methylbutanoate	56.6 ^a^	±2. 9	56.2 ^a^	±3.5	53.2 ^a^	±7.5	0.685
Ethyl hexanoate	491.2 ^c^	±18.2	357.1 ^b^	±32.5	300.4 ^a^	±29.1	0.000
Ethyl octanoate	245.1 ^b^	±7.7	153.0 ^a^	±37.2	138.9 ^a^	±14.4	0.003
Ethyl decanoate	18.8 ^a^	±1.7	16.0 ^a^	±6.6	12.1 ^a^	±3.1	0.246
Ethyl lactate	3704.2 ^b^	±158.1	3656.8 ^b^	±99.3	3194.4 ^a^	±352.9	0.065
**Fatty Acids**							
3-Methylbutanoic acid	706.1 ^b^	±115.7	306.1 ^a^	±8.8	448.0 ^ab^	±194.8	0.025
Hexanoic acid	3753.0 ^b^	±77.1	1833.3 ^a^	±156.2	1878.8 ^a^	±235.0	<0.0001
Octanoic acid	2901.9 ^b^	±140.7	1288.8 ^a^	±183.5	1405.4 ^a^	±166.7	<0.0001
**Terpenes**							
*cis*-Linalool oxide	7.85 ^a^	±0.53	8.57 ^ab^	±0.59	9.60 ^b^	±0.66	0.031
*trans*-Linalool oxide	4.84 ^a^	±0.30	6.07 ^b^	±0.18	6.06 ^b^	±0.28	0.002
Linalool	15.8 ^a^	±3.17	23.1 ^a^	±3.1	18.80 a	±3.3	0.142
Terpinen-1-ol	<LOQ		<LOQ		<LOQ		
Terpinen-4-ol	11.7 ^a^	±2.7	17.5 ^b^	±2.0	13.4 ^a,b^	±1.8	0.044
Ho-trienol	0.013 ^a^	±0.006	0.018 ^a^	±0.003	0.022 ^a^	±0.003	0.115
α-Terpineol	34.2 ^a^	±3.3	37.5 ^a^	±3.9	34.6 ^a^	±3.3	0.511
Nerol	1.47 ^a^	±0.57	1.33 ^a^	±0.75	2.29 ^a^	±0.06	0.147
Geraniol	2.97 ^a^	±0.14	4.31 ^a^	±1.99	2.64 ^a^	±1.02	0.319
β-Citronellol	2.71 ^a^	±2.37	7.92 ^b^	±1.60	4.38 ^a^	±0.99	0.027
p-Menthane-1.8-diol	0.417 ^b^	±0.060	0.214 ^a^	±0.069	0.404 ^b^	±0.054	0.011
α-Phellandrene	0.802 ^a^	±0.023	0.518 ^a^	±0.237	0.620 ^a^	±0.076	0.218
1,4-Cineole	0.202 ^a^	±0.169	0.286 ^a^	±0.041	0.323 ^a^	±0.045	0.398
1,8-Cineole	0.188 ^a^	±0.032	0.307 ^a^	±0.024	0.362 ^a^	±0.018	0.341
Limonene	0.408 ^a^	±0.178	0.497 ^a^	±0.026	0.430 ^a^	±0.038	0.596
γ-Terpinen	1.01 ^a^	±0.13	0.294 ^a^	±0.131	0.992 ^a^	±0.040	0.664
p-Cymene	0.162 ^a^	±0.061	0.252 ^b^	±0.016	0.155 ^a^	±0.018	0.034
Terpinolene	0.115 ^a^	±0.036	0.175 ^b^	±0.015	0.098 ^a^	±0.013	0.017
**Norisoprenoids**							
β-Damascenone	3.49 ^a^	±0.05	3.08 ^a^	±0.18	3.58 ^a^	±0.10	0.418
α-Ionone	0.282 ^a^	±0.128	0.448 ^a^	±0.099	0.480 ^a^	±0.287	0.441
α-Ionol	0.130 ^a^	±0.0317	0.140 ^a^	±0.094	0.295 ^a^	±0.096	0.554
Vitispirane	5.07 ^a^	±4.66	8.33 ^a^	±0.51	7.72 ^a^	±0.89	0.367
TPB	0.080 ^b^	±0.013	0.049 ^a^	±0.006	0.055 ^a^	±0.008	0.015
TDN	4.25 ^a^	±1.34	4.20 ^a^	±0.68	4.50 ^a^	±0.35	0.906
3-oxo-α-Ionol	17.6 ^a,b^	±4.2	11.6 ^a^	±2.8	20.7 ^b^	±3.4	0.080
**Benzenoids**							
4-Ethyl guaiacol	0.190 ^a^	±0.049	0.295 ^a^	±0.152	0.153 ^a^	±0.193	0.500
4-Vinyl guaiacol	15.5 ^a^	±4.4	21.3 ^a^	±0.7	20.2 ^a^	±5.1	0.235
2,6-Dimethoxyphenol	0.078 ^a^	±0.014	0.110 ^a^	±0.029	0.208 ^a^	±0.043	0.220
Benzyl Alcohol	153.8 ^a^	±27.3	275.0 ^c^	±12.1	215.2 ^b^	±16.3	0.001
Vanillin	5.36 ^a^	±1.04	7.26 ^b^	±0.37	6.39 ^ab^	±0.61	0.050
Methyl vanillate	4.83 ^a^	±0.50	5.96 ^b^	±0.37	5.79 ^b^	±0.34	0.030
Ethyl vanillate	34.4 ^a^	±6.0	56.7 ^b^	±3.7	69.7 ^b^	±3.4	0.012

Values in the same row with different letters indicate statistically significant differences (*p* < 0.05). <LOQ: Value below the limit of quantification.

**Table 4:** Concentration (µg/L) of bound compounds in aged wine samples. Mean, standard deviation (SD) and ANOVA.

	Control		Fruttaio		On-Vine		
Compounds	Mean	SD	Mean	SD	Mean	SD	*p*-Value
**Alcohols**							
Phenylethyl alcohol	121.4 ^ab^	±7.8	96.2 ^a^	±21.9	146.9 ^b^	±9.4	0.015
**C6 alcohols**							
1-Hexanol	84.6 ^a^	±6.7	84.8 ^a^	±14.0	82.8 ^a^	±7.4	0.965
*trans*-3-Hexen-1-ol	0.588 ^a^	±0.035	0.852 ^b^	±0.163	0.750 ^ab^	±0.065	0.054
*cis*-3-Hexen-1-ol	22.6 ^b^	±1.6	11.2 ^a^	±5.2	13.8 ^a^	±0.8	0.011
**Terpenes**							
*cis*-Linalooloxide	2.23 ^a^	±0.26	2.41 ^a^	±0.46	2.79 ^a^	±0.32	0.229
*trans*-Linalooloxide	2.83 ^a^	±0.74	3.05 ^a^	±0.71	3.68 ^a^	±0.64	0.366
Linalool	<LOQ		<LOQ		<LOQ		
Terpinen-4-ol	<LOQ		<LOQ		<LOQ		
α-Terpineol	0.182 ^a^	±0.029	0.977 ^b^	±0.253	0.977 ^b^	±0.140	0.009
β-Citronellol	0.175 ^a^	±0.004	1.19 ^b^	±0.20	0.000 ^a^	±0.096	<0.0001
Nerol	3.60 ^a^	±0.60	4.68 ^a^	±0.20	3.95 ^a^	±0.73	0.128
Geraniol	4.08 ^a^	±1.03	6.95 ^b^	±0.56	5.20 ^ab^	±1.63	0.060
**Norisoprenoids**							
3-oxo-α-Ionol	2.59 ^a^	±0.22	2.11 ^a^	±0.37	3.17 ^b^	±0.24	0.011
α-Ionol	<LOQ		<LOQ		<LOQ		
**Benzenoids**							
Vanillin	3.76 ^a^	±0.07	3.61 ^a^	±0.30	3.76 ^a^	±0.16	0.595
Methyl vanillate	3.94 ^a^	±0.09	5.22 ^b^	±0.31	5.02 ^b^	±0.28	0.001
Ethyl vanillate	0.317 ^a^	±0.132	0.737 ^a^	±0.135	0.668 ^a^	±0.209	0.134
Benzyl Alcohol	130.5 ^b^	±6.2	65.7 ^a^	±3.4	105.9 ^ab^	±8.5	0.023

Values in the same row with different letters indicate statistically significant differences (*p* < 0.05). <LOQ: Value below the limit of quantification.

**Table 5:** Composition of Corvina wines made from not withered grapes, withered on-vine and withered in “fruttaio”.

	Brix	pH	Alcohol % vol
Control	21	2.96 ± 0.05	13.6 ± 0.2
On-vine	23.2	2.92 ± 0.08	15.0 ± 0.2
Fruttaio	23.2	3.03 ± 0.06	15.2 ± 0.2

**Table 6:** Retention indices, quantification ions of studied compounds.

	Method ^1^	LRI ^1^	Identification ^2^	Quantitation Ion *m*/*z*	Qualifier Ions *m*/*z*	LOD (µg/L)	LOQ (µg/L)
1-Butanol	a	1159	RS	56	55	0.02	0.06
2-Butanol	a	1020	RS	59		0.20	0.6
1-Pentanol	a	1256	RS	55	56, 57, 70	0.04	0.11
Isoamyl alcohol	a	1220	RS	57	55, 56, 70	0.02	0.06
Phenylethyl Alcohols	a	1920	RS	91	65, 92, 122	1.95	5.84
1-Hexanol	a	1316	RS	56	55, 69	0.76	2.27
*trans*-3-Hexen-1-ol	a	1379	RS	67	55, 69, 82	0.40	1.21
*cis*-3-Hexen-1-ol	a	1391	RS	68	55, 69, 83	1.23	3.68
Ethyl acetate	b	895	RS	88	61, 70	0.5	1.58
Isoamyl acetate	a	1125	RS	70	55, 60, 87	0.03	0.1
n-Hexyl acetate	a	1271	RS	56	55, 61, 84	0.03	0.1
Ethyl 3-methyl butanoate	a	1069	RS	88	57, 60, 85	0.30	0.9
Ethyl butanoate	a	1032	RS	71	88	0.01	0.04
Ethyl hexanoate	a	1240	RS	88	60, 99	5.82	17.47
Ethyl octanoate	a	1430	RS	88	57, 100, 127	0.54	1.63
Ethyl decanoate	a	1640	RS	88	71, 101, 155	0.16	0.49
Ethyl lactate	a	1340	RS	75	88, 90	2.1	6.3
3-Methylbutanoic acid	a	1667	RS	60	87	0.17	0.52
Hexanoic acid	a	1839	RS	60	73, 87	0.15	0.46
Octanoic acid	a	2071	RS	60	73, 101, 115	0.00	0.01
*cis*-Linalooloxide	b	1437	RS	59	111, 94	0.02	0.07
*trans*-Linalooloxide	b	1469	RS	59	111, 94	0.02	0.07
Linalool	b	1547	RS	71	121, 93	0.08	0.25
Geraniol	b	1860	RS	93	123, 121, 105	0.06	0.2
β-Citronellol	b	1771	RS	69	82, 81, 67	0.07	0.21
α-Terpineol	b	1701	RS	136	121, 93, 59	0.23	0.7
α-Phellandrene	b	1180	RS	93	136, 91	0.001	0.003
γ-Terpinen	b	1188	RS	121	93, 126	0.03	0.1
Limonene	b	1198	RS	136	139, 125, 111	0.03	0.1
1,4-Cineole	b	1186	RS	154	139, 111, 108	0.003	0.011
1,8-Cineole	b	1217	RS	154	139, 111, 108	0.003	0.011
p-Cymene	b	1271	RS	119	134, 91	0.02	0.06
Terpinolene	b	1283	RS	121	136, 93	0.03	0.09
Terpinen-1-ol	b	1581	LRI MS	136	121, 81	-	-
Terpinen-4-ol	b	1614	RS	71	111, 93, 86	0.02	0.05
p-Menthane-1,8-diol	a	2250	RS	96	88, 139	0.03	0.09
Ho-trienol	b	1585	LRI MS	82	67, 71	-	-
Nerol	b	1812	RS	93	121, 84, 69	0.04	0.12
β-Damascenone	b	1825	RS	69	190, 121, 105	0.01	0.03
α-Ionone	b	1853	RS	121	136, 192	0.02	0.06
α-Ionol	b	1925	RS	95	123, 138	0.04	0.12
3-Oxo-α-ionol	a	2555	LRI MS	108	152	-	-
Vitispirane	b	1523	LRI MS	192	177, 93	-	-
TPB	b	1828	LRI MS	172	157, 142	-	-
TDN	b	1745	LRI MS	157	172, 142	-	-
Benzyl Alcohols	a	1874	RS	106	105, 77, 51	0.03	0.1
Vanillin	a	2572	RS	151	81, 152, 109	0.01	0.02
4-Ethyl guaiacol	a	1988	RS	137	122, 152	0.03	0.09
4-Vinyl guaiacol	a	2212	RS	150	107, 135	0.07	0.21
Ethyl vanillate	a	2665	RS	151	168, 196	2.36	7.09
Methyl vanillate	a	2630	RS	151	123, 182	0.97	2.91
2,6-Dimethoxyphenol	a	2270	RS	154	95, 111, 139	0.01	0.03

^1^ Extraction method: a (SPE) and b (SPME) ^2^ Linear Retention Index (LRI) were determined on DB-WAX polar column, as described by van Den Dool and Kratz (1963) [52]. RS identified using reference standard; LRI MS tentatively identified by comparing the Linear Retention Index and mass spectra with those of literature.
